# The complete chloroplast genome sequence of *Ostericum koreanum* (Apiaceae)

**DOI:** 10.1080/23802359.2016.1157772

**Published:** 2016-03-29

**Authors:** Sun A. Choi, Woo Kyu Lee, Yeji Kim, Kyu-Yeob Kim, Jong Hwan Kim, Rack Seon Seong

**Affiliations:** a Herbal Medicine Research Division, National Institute of Food and Drug Safety Evaluation, Ministry of Food and Drug Safety, Cheongju-si, Chungcheongbuk-do, Republic of Korea;; b Herbal Medicine Policy Division, Ministry of Food and Drug Safety, Cheongju-si, Chungcheongbuk-do, Republic of Korea

**Keywords:** *Angelica reflexa*, chloroplast, genome sequencing, *Ostericum koreanum*, taxonomic status

## Abstract

*Ostericum koreanum* Kitagawa is an important herbal medicine, whose taxonomic status has been changed to *Angelica reflexa* as a new species. This study generated the complete chloroplast genome sequence of *O. koreanum*, and reconsidered its molecular taxonomic status in *Angelic*a by comparing it with related species. The length of the complete chloroplast genome was 147,282 bp, and there were four structures that included the large single copy region (93,185 bp), the small single copy region (17,663 bp) and the duplicated inverted regions (18,217 bp of each). Based on its phylogenetic trees, *O. koreanum* was grouped by high bootstrap value with the *Angelica* species. This result proved that *O. koreanum* is included in *Angelica*. Therefore, this chloroplast genome data generated for the first time a valuable genetic resource for the discrimination of herbal materials, phylogeny, and evolution.

*Ostericum koreanum* Kitagawa, has been used as a beneficial herbal medicinal plant with rejuvenating effects, belongs to the family Apiaceae (Kim et al. 2014). This species has been known as a controversial taxon, whose classification has been changed several times. Maximowicz (1886) reported a new species as *Angelica koreana*, and this species was then transferred to the genus *Ostericum* and *O. grosseserrata* by Kitagawa (1936, 1971), respectively. Recently, however, Lee et al. (2013) reported that *O. koreanum* was reclassified to *Angelica reflexa* as a new species. In this study, we determined the complete chloroplast genome of *O. koreanum* for the first time, and estimated its molecular taxonomic status in *Angelica*.

The plant sample of *O. koreanum* was collected from the herbal garden of the National Center for Herbal Medicine Resources in Korea. The genomic DNA was extracted from dried leaf and assembled to Illumina paired-end library. Genomic sequencing was performed using Illumina Hiseq 2000 instrument (Illumina, San Diego, CA), and assembled by CLC genomic assembler (v. beta 4.6, CLC Inc., Rarhus, Denmark). The gene annotation was conducted to DOGMA (http://dogma.ccbb.utexas.edu/) and manual curation using BLAST. The voucher specimen was preserved in the National Center for Herbal Medicine Resources.

The total size of the assembled chloroplast genome of *O. koreanum* was 147,282 bp (accession no. KT852844), and the genome reads were 215× of average coverage. The genome structure consisted of the typical four parts, which were large single copy region (LSC) of 93,185 bp, small single copy region (SSC) of 17,663 bp and a pair of inverted regions (IR) of 18,217 bp each. The GC content was 37.54%. The genome had a total of 113 genes, including 80 protein-coding genes among which nine (*ndhB*, *orf42*, *orf56*, *rps7*, *rps12*, *ycf1*, *ycf2*, *ycf15* and *ycf68*) were duplicated in the IR region. There were 29 tRNA genes distributed throughout the genome, with 22 in the LSC, one in the SSC and six in the individual IRs. There were four rRNA, the *rrn4.5*, *rrn5*, *rrn16* and *rrn23* genes, distributed in only the IR regions.

To determine the taxonomic status of *O. koreanum* within *Angelica*, phylogenetic analyses based on maximum parsimony (MP) and maximum likelihood (ML) were performed from the genome sequences of 11 taxa, consisting of genera *Angelica, Ligusticum, Glehnia, Peucedanum* and *Saposhnikovia*. These sequences were aligned using MAFFT (http://mafft.cbrc.jp/alignment/software/). The MP tree was constructed by 10 random additions, tree-bisection-reconnection (TBR), and 1000 bootstrap replications in MEGA6 (Tamura et al. 2013). The ML tree was produced by GARLI Web Services (www.molecularevolution.org), with general time reversible parameters based on gamma distribution and 1000 replicates.

Both the MP and the ML trees were similar, as indicated by their bootstrap support values on the branches and topology of trees (Figure 1). In MP tree, *O. koreanum* showed monophyletic relation with *Angelica* species, supported by high statistical values of nearly 100%, and in ML tree *O. koreanum* showed a close relationship with the *Angelica* species. Therefore, our data of complete genomic sequences proved the study by Lee et al. (2013) that *O. koreanum* is included in genus *Angelica.* This chloroplast genome data could be supported to discriminate between genuine species for herbal medicine and adulterations, to analyze the relation of closely related taxa, and to study phylogeny and evolution.

**Figure 1.**
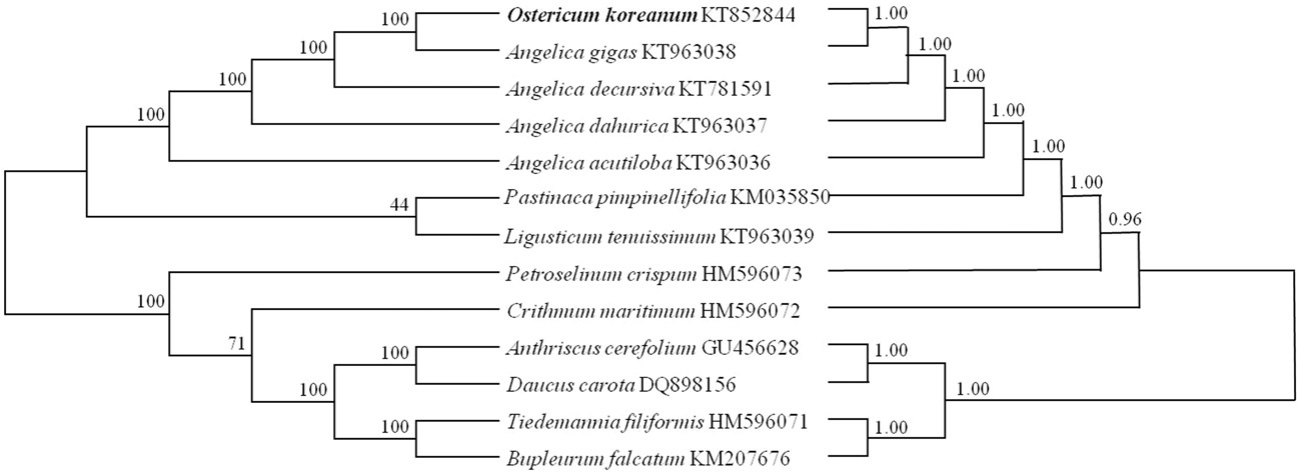
Phylogenetic trees based on maximum parsimony (left) and maximum likelihood (right) analysis. The topologies of the two trees are congruent with each other. The numbers above the branches are the bootstrap statistics values of maximum parsimony and maximum likelihood, respectively.
